# How to design a Q-sample: A seven-step approach based on interview data

**DOI:** 10.3205/zma001802

**Published:** 2026-01-15

**Authors:** Nana Jedlicska, Sabrina Lichtenberg, Pascal O. Berberat, Kristina Schick

**Affiliations:** 1 Technical University of Munich, TUM School of Medicine & Health, Department Clinical Medicine, TUM Medical Education Center, Munich, Germany; 2 TU Dresden University of Technology, Medical Faculty and University Hospital Carl Gustav Carus, Institute of Medical Education, Dresden, Germany

**Keywords:** Q-methodology, measuring subjectivity, physicians’ role expectations, designing Q-sample, editing Q-sample

## Abstract

In recent decades, medical education research has increasingly investigated the subjectivity and viewpoints of (pre-service) healthcare professionals. A promising approach for exploring subjectivity is Q-methodology (Q). Q, which combines qualitative and quantitative methods, involves a card-sorting process in which participants are asked to sort statements into a (normal distribution) grid according to their preferences. Similar sorting patterns are then summarized into profiles and described narratively. A central element of this process is the design of the Q-sample – a set of statements representing a wide range of opinions, beliefs, or perspectives on the subject of study. The Q-sample is, therefore, critical for the success of a Q-study and requires precise development steps. Currently, these steps are only preliminarily described in the literature. The present paper addresses this gap by defining a seven-step approach to Q-sample design based on interview data. It offers a systematic and methodological approach that captures the diversity of viewpoints on a particular research topic. Building on a previous qualitative study, it demonstrates how to translate interview data into a Q-sample while ensuring coverage and balance through the use of a mapping technique. The paper also addresses the significance of editing and how to preserve the everyday language of participants when modifying the Q-sample to facilitate self-reference. A comprehensive overview of the criteria for designing a Q-sample is provided. Practical recommendations for selecting a Q-sample and implementing Q-methodology in medical education are offered, and potential challenges are discussed in detail.

## Abstract (de)

In den letzten Jahrzehnten hat die Forschung zur medizinischen Ausbildung zunehmend die Subjektivität und die Perspektiven von (angehenden) Gesundheitsfachkräften untersucht. Ein vielversprechender Ansatz zur Erforschung von Subjektivität ist die Q-Methode (Q). Die Q-Methode kombiniert qualitative und quantitative Forschungsansätze. Die Teilnehmenden sortieren dabei Aussagen entsprechend ihrer Präferenzen in ein (normalverteiltes) Raster. Ähnliche Sortiermuster werden anschließend zu Profilen zusammengefasst und narrativ beschrieben. Ein zentrales Element dieses Verfahrens ist die Gestaltung des Q-Samples – eine Zusammenstellung von Aussagen, die ein breites Spektrum von Meinungen, Überzeugungen und Perspektiven zum Untersuchungsgegenstand repräsentieren. Das Q-Sample ist daher entscheidend für den Erfolg einer Q-Studie und erfordert klar definierte Entwicklungsschritte. Derzeit sind diese Schritte in der Literatur lediglich ansatzweise beschrieben. Die vorliegende Arbeit schließt diese Lücke, indem sie ein siebenstufiges Verfahren für das Design eines Q-Samples auf der Grundlage von Interviewdaten beschreibt. Es wird ein systematischer und methodischer Ansatz präsentiert, der die vielfältigen Standpunkte zu einem bestimmten Forschungsthema erfasst. Aufbauend auf einer früheren qualitativen Studie wird aufgezeigt, wie Interviewdaten in ein Q-Sample überführt werden können, wobei durch den Einsatz einer Mapping-Technik thematische Abdeckung und Ausgewogenheit sichergestellt werden. Der Beitrag befasst sich auch mit der Bedeutung redaktioneller Überarbeitung und der Frage, wie die Alltagssprache der Teilnehmenden bei der Modifizierung des Q-Samples erhalten werden kann, um Selbstbezug anzuregen. Es wird ein umfassender Überblick über die Kriterien zur Gestaltung eines Q-Samples gegeben. Zudem werden praxisnahe Empfehlungen für die Auswahl eines Q-Samples und die Anwendung der Q-Methode in der medizinischen Ausbildung ausgesprochen und potenzielle Herausforderungen werden im Detail diskutiert.

## Introduction to Q-methodology

A growing area of interest in healthcare education research is the exploration of subjectivity among healthcare professionals, including their individual experiences, attitudes, and role expectations [1], [2], [3], [4]. Subjectivity refers to individuals’ perceptions of their environment, shaped by their experiences and attitudes (self-reference; [5]). A promising approach for investigating subjectivity is Q-methodology (Q; [6], [7], [8]). Q is characterized by its less-confrontational nature and, therefore, offers a holistic approach to understanding different perspectives on complex or delicate topics [9]. In line with Watts and Stenner [10], we conceive of Q as a method for assessing attitudes as subjective recognitions of the environment (constructivism), and as being influenced by one’s social and sociological background (constructionism) [10]. Constructivism assumes that people perceive and interpret the world based on their values, experiences, attitudes, and ideas, forming an individual perspective. For example, medical students bring experiences and attitudes from their primary socialization (childhood, adolescence, social milieu) into their clinical clerkships. These perspectives, often described as seeing the world through one’s own “glasses”, can also change due to external influences and new experiences [10], [11]. However, constructionism emphasizes the role of social interaction in shaping viewpoints. It suggests that perspectives are not only individually constructed but also shaped by group membership and social roles. For example, the attitudes of (prospective) doctors toward (dealing with) dying and death may differ from those of relatives. A person may adopt certain views depending on their social role: as a doctor, they may take on a professional perspective, while as a relative, their viewpoint may be shaped by personal emotions and experiences [10], [12].

In addition to constructivism and constructionism, the interactionist role theory, as a sociological and social-psychological framework, provides valuable conceptual insights into the development of subjective perceptions of one’s environment. It places the individual in interrelation with the social structure and emphasizes the possibility for individuals to shape their roles. Role expectations are understood as a catalogue of possible behaviours. They serve as interpretive patterns of social reality, making appearances recognizable and classifiable as “typical”. Role expectations are, therefore, not simply internalized and routinely enacted. Instead, individuals are expected to select from actions considered typical for their role and curate their behaviour themselves (role-making). The role is viewed as a dynamic phenomenon, negotiated in the interaction process between partners and shaped individually [13]. Focusing on an interactionist understanding of role in the present study allows us to uncover the common role expectations of physicians. Further, this perspective enables us to explore how medical trainees individually configure their roles.

To uncover different patterns of perception across individuals, Q combines qualitative and quantitative research approaches. Since Q requires participants to consider various aspects of a topic in relation to one another to adopt and express an attitude toward these aspects, it facilitates self-reflexivity [14]. Thus, besides capturing subjectivity, Q also contributes to the formation of subjective perceptions about a topic. Additional strengths of Q are the usability of its results and the rigor and practical nature of the methodology [11]. Q therefore promotes the construction of meaning while reducing the impact of prior assumptions and potential researcher bias, and simultaneously ensures validity [15]. It’s requirement for only a small number of participants further increases its practicality [5]. Through Q, attitudes can be uncovered across different interest groups in a time- and resource-efficient manner [5].

The viewpoints of different groups are of particular interest in medical education, including patients, students, teachers, and nurses. In medical education and health professions research, Q studies address numerous topics relevant to the field, aiming to investigate attitudinal patterns and opinions of (future) health professionals [1], [4], [9]. Examples include studies on the professional identities of health professionals [16], [17], preferences regarding residency training [18], attitude changes towards medical communication [2], or perspectives on simulation training [19], [20]. 

To detect these viewpoints, a concourse must be defined. The concourse represents the shared understanding of the topic under discussion [5]. It can be constructed using various data sources. The concourse may be based on perceptions, opinions, terminologies, and definitions that exist within society and can be gathered from newspapers, social media content, or theories derived from research (e.g., interview studies and focus groups) [9]. The aim of integrating the various sources is to capture as complete a picture of the topic as possible, from which the Q-sample is then derived [5], [10]. Based on the concourse, items are developed in the form of statements “to provide a comprehensive but manageable representation of the concourse […]” [5]. These items make up the *Q-sample*. Participants are asked to arrange the statements of the Q-sample into either a normal distribution (forced) or free distribution (unforced) grid, following sorting instructions such as “Sort the items from those with which you *most agree* (+3) to those with which you *most disagree* (-3)” [5]. This card-sorting process yields a Q-sort for each participant, in which each statement occupies a specific position within the grid. The individual Q-sorts are then analyzed using the *Q-method*. The Q-method aims to cluster similar Q-sorts (i.e., similar arrangements of statements) into attitudinal profiles through by-person (Q-) factor analysis. These profiles differ in how statements are arranged. Specifically, the positions of statements in these profiles are interpreted narratively by comparing their arrangement both within a single profile and across profiles (for a detailed methodological description, see e.g. Watts and Stenner, 2012 [10], McKeown and Thomas, 2013 [5]). 

## The aim of the present paper

Given the increasing popularity of Q, several recent studies have focused on developing a concourse and selecting a Q-sample [15], [21], [22], [23], [24], [25], [26], [27]. Many of these studies refer to *Fisher’s Design of Experiments* as a theoretical framework for selecting a Q-sample in order to ensure that the selection comprehensively represents the concourse [21], [22], [23], [24]. Kirschbaum et al. propose a three-step approach for generating a Q-sample, incorporating a theoretical framework and the Delphi technique with expert panels [15]. Paige and Morin describe an iterative process for Q-sample development consisting of four steps, emphasizing the importance of a structured and methodological approach [25]. These studies address various aspects of Q-sample development. For example, Mohr highlights the importance of linguistic and contextual considerations and emphasizes editing statements to reflect the specific characteristics of the respective language [26]. Ramlo demonstrates how to develop the concourse and select a Q-sample using ChatGPT [22]. Lee describes software programs that assist Q researchers in generating concourse statements from different information sources and refining them into a Q-sample [28]. However, we identified limitations in the reporting of Q-sample development [9], [22], [25]. In particular, there is insufficient guidance on systematically extracting themes from diverse data sources, especially interview data. A holistic overview of the criteria for designing a solid Q-sample is also lacking. This paper addresses these gaps by outlining a systematic approach to building a Q-sample derived from interview data, focusing on the following aspects:

1. the application of a mapping technique to ensure coverage and balance;
2. the processing of the Q-sample while preserving participants’ everyday language [21]; and
3. the provision of a comprehensive overview of the criteria for selecting and editing a given Q-sample.

## Methodological approach

### Designing a Q-sample

The present study builds on our previous research, in which we investigated final-year medical students’ and residents’ understandings of their roles as (future) physicians in dealing with dying and death (hereafter referred to as the “interview study” [3], [29]. In the interview study, 18 semi-structured interviews were conducted using an interview guide [3], [29]. These interviews explored medical students’ and residents’ formative experiences regarding dying and death, their attitudes toward death, and their understanding of their roles as (future) physicians in caring for dying patients [3], [29]. Approval for the Q-study was obtained from the ethics committee of the faculty of medicine of the Technical University of Munich (project number: 489/21S-NP).

The Q-sample design was preceded by the formulation of a research question. The research question shapes the nature and structure of the Q-sample and guides the sorting process [30]. To define a clearly stated, concise, and focused research question containing only a single proposition [10], we agreed on the following: 

“What patterns can be identified in physicians’ perceptions about their role in dealing with dying patients and death?”

A Q-sample design aims to develop a “workable number” of statements [31] that ensure good coverage and balance in relation to the research question and broadly represent the concourse on the subject [21], [30]. In the Q-context, balance means covering all relevant aspects of a topic to represent a broad range of viewpoints and opinions in the Q-sample. It is crucial to avoid a one-sided presentation and instead capture the full diversity of views. Balance does not require creating the same number of statements for each topic; rather, the number of statements should reflect their significance in relation to the overarching research question [10]. Key requirements for the individual statements include relevance to the study topic, clarity, unambiguous meaning, conciseness, distinguishability, and a consistent format [7], [10], [31], [32], [33], [34]. Additionally, the statements should be self-referential, enabling participants to relate them to their own experiences, beliefs, or feelings, thereby facilitating a more personal evaluation [32].

In the following, we describe a seven-step approach to developing a Q-sample that effectively addresses the research question [30]. Table 1 (Tab. 1) provides an overview of the steps in the Q-sample design.

### Populate the concourse

#### Step 1 – Inductive identification of themes and sub-themes in the data

**Figure 1:**
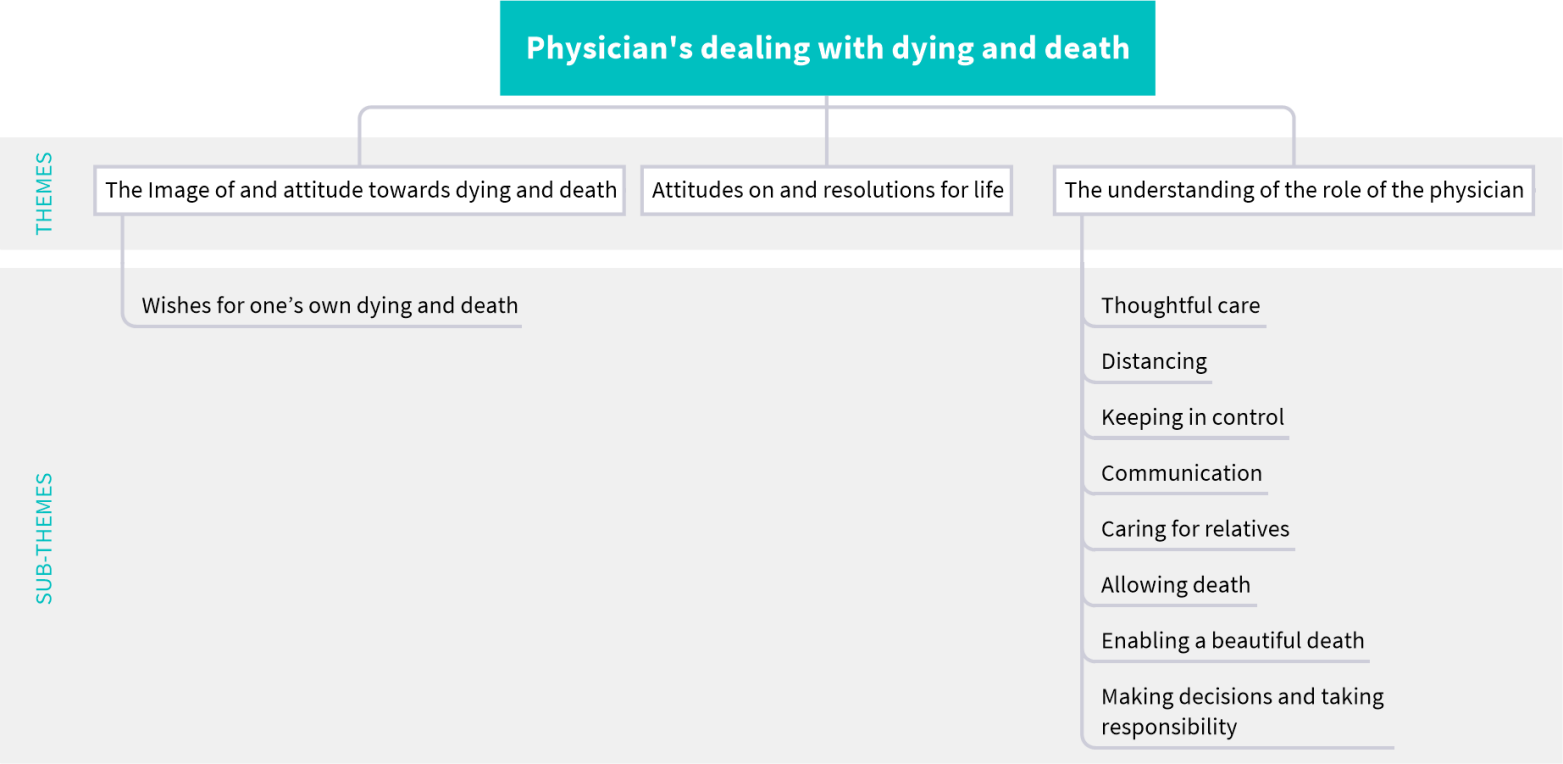
An overview of themes and sub-themes

Developing a Q-sample involves constructing a concourse, a set of items that broadly represent the range of opinions and communications on the subject [10], [21], [23], [35]. For our Q-study, we populated the concourse based on the themes and sub-themes identified in our interview study by applying Schreier’s qualitative content analysis approach (QCA; [3], [29], [36]). The (sub-) themes were developed inductively and then compared with, and integrated into, the current state of research and existing theories regarding how health professionals deal with dying and death. Our concourse included three main themes with nine sub-themes in total (see figure 1 (Fig. 1)). For detailed information about the qualitative analysis, see Jedlicska et al., 2024 [29].

#### Step 2 – Elaborating and mapping central perceptions across themes and sub-themes

**Figure 2:**
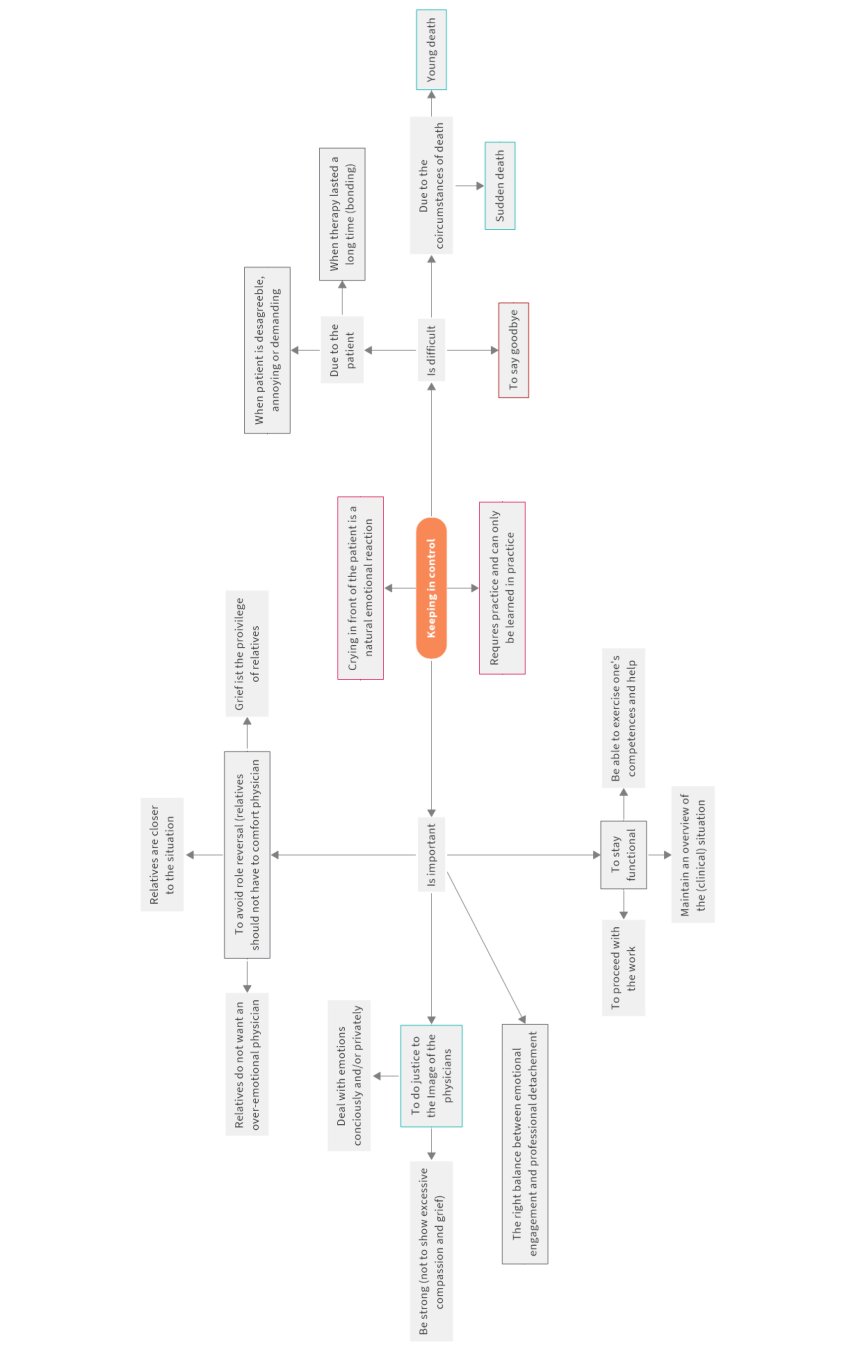
Concept map keeping in control Note: Orange=sub-theme, grey=perceptions and aspects of perceptions, boxed black=perceptions covered by other sub-themes, boxed red=disregarded perceptions due to a lack of relevance, boxed green=perceptions that informed statements

Next, the central perceptions for each theme and sub-theme are elaborated. These perceptions form the basis for statements that capture diverse viewpoints, opinions, and communications on the topic [9]. A concept map can be used to represent the identified perceptions corresponding to the themes and sub-themes (see figure 2 (Fig. 2)). SL and NJ carried out the mapping process collaboratively. To minimize researcher bias and ensure validity, each researcher initially developed a preliminary concept map for each theme and sub-theme, based on interview data and memos written during the interview study. For this purpose, each researcher selected key perceptions (concepts) from the interviews and memos, grouped similar perceptions, and organized them hierarchically (from most general to most specific) while ensuring that the relationships between the perceptions were accurately depicted [37]. At the end of this step, two concept maps were created for each theme and sub-theme. In the subsequent step, SL and NJ reviewed both maps for each theme and sub-theme and merged them into a single map. During this process, the researchers critically discussed the relevance of each perception, examined their interrelationships, and refined their hierarchical structure. The concept maps were reviewed for completeness and clarity. To ensure comprehensive coverage of the study topic, the researchers also consulted relevant literature and incorporated additional perceptions from prior research. The aim was to capture both the breadth and depth of each theme and sub-theme [32] and to fully represent the diversity of viewpoints on the research topic [30]. Moreover, the mapping technique provided us with an overview of the perceptions expressed throughout the interviews and helped uncover and illustrate the connections between them, thereby deepening the understanding of each theme and sub-theme [38]. In the end, we had 11 maps that covered all themes and sub-themes in detail. These maps formed the concourse. Figure 2 (Fig. 2) shows an example of a map for the sub-theme “keeping in control”.

### Constructing the Q-sample

#### Step 3 – Selecting relevant perceptions

In the third step, the relevant perceptions should be identified for inclusion in the preliminary Q-sample. To select the perceptions appropriately, a reasonable level of detail was maintained to ensure coverage and balance in the Q-sample, while also considering manageability in terms of the total number of statements [10]. The number of statements should be kept as low as possible to avoid overwhelming the participants [30]. We reviewed and discussed all 11 maps individually and selected the perceptions to be included in the Q-sample. Our decisions were based on the following inclusion criteria:

1. the relevance of the topic in the interviews and to the interviewees;
2. consistency with previous research findings, to ensure comprehensiveness; and
3. the diversity of opinions.

Exclusion criteria were as follows:

1. lack of coverage or overlapping perceptions; and
2. a narrow or overly specific focus (e.g., infant death).

This step resulted in 84 perceptions considered relevant to the research question.

#### Step 4 – Assembling the preliminary Q-sample 

In the following step, the selected perceptions are compiled into statements, resulting in a preliminary Q-sample. We decided to design the Q-sample based on quotations. Personal language (i.e., using terms from participants’ everyday language) ensured that the statements resonated with their personal experiences and life contexts [39], thereby enhancing their comprehensibility [23], [32]. Furthermore, statements were formulated in the first person to address participants directly, making it easier for them to empathize with the statements and thereby facilitating the rank-ordering process [32]. Each statement focused on a single idea (not double-barreled) [15].

Initially, corresponding quotes were identified for each perception in the interview data. Two approaches were applied to create the Q-sample. First, original quotations were edited by removing filler words and subordinate clauses and shortening them to their core messages, reducing complexity and improving statement manageability [15]. These edited quotes were then converted into statements. For example, for the sub-theme “communication”, the original quote “that you clearly state how the medical situation is” was revised to: “As a physician, I have to speak openly with the patient about their medical situation”. Second, when quotations did not accurately reflect the central idea, core elements were identified and added to the statements. These core elements were the sentence parts that conveyed key messages recurring across interviews, often with only slight variations. This made identifying core elements intuitive. If multiple core elements are found for a single perception, the research group should discuss the options and reach a consensus on a selected option. For example, in the sub-theme “allowing death”, the expressions “to find the point” and “to find the right time” both appeared as central and recurring issues. We chose “find the point” due to its higher level of abstraction, which still implied the time aspect of allowing death. The final statement became: “As a physician, I have to find the point where I let patients go”. 

**Figure 3:**
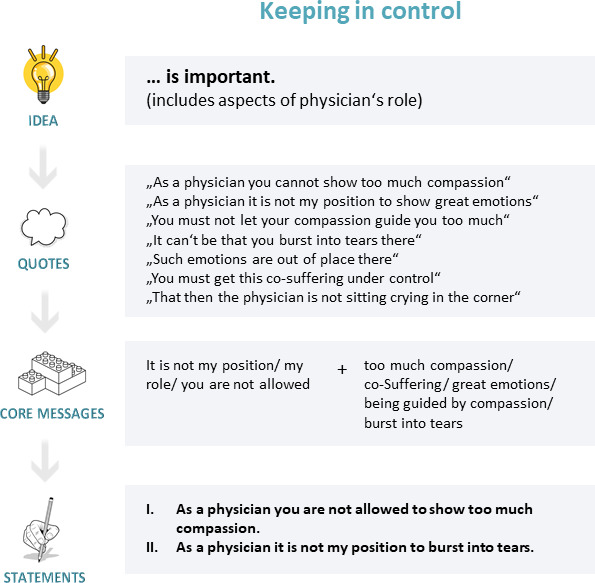
Processing quotes into statements

While working with the quotations, further overlaps or inconsistencies became apparent, prompting further refinement in line with the criteria described in step 3. Thus, the Q-sample design is an iterative or even recursive process that involves moving back and forth between individual steps. During statement selection, explanatory content in statements should be avoided, allowing participants to assign their own meaning to the statements [25]. A key consideration in developing a Q-sample is how to balance the number of statements per theme. Q-methodology discusses two approaches: the structured and unstructured approaches. The structured approach, similar to a quantitative questionnaire design, ensures an equal number of statements per theme. In contrast, the unstructured approach aligns with qualitative research, aiming to represent the concourse as a whole. Here, the focus is not on having an equal number of statements per topic; the number of statements varies based on their relevance. This ensures a balanced Q-sample accurately reflecting the significance of statements within the overall theme. Our interview study revealed themes and subthemes of differing importance to the interviewees (in terms of scope and detail of the (sub-)themes). To reflect these different emphases, we selected an unequal number of statements for each theme and sub-theme in our Q-sample (unstructured approach), mirroring the relevance of the different (sub-)themes in the interview study. As a result of step 4, we condensed the 84 perceptions into a preliminary Q-sample comprising 62 statements (see figure 3 (Fig. 3)).

#### Step 5 – Refining the preliminary Q-sample

Next, the catalog is refined by removing duplicates, redundancies, and reversed or opposing statements [31]. The statements should be critically reviewed regarding intelligibility, simplicity, and brevity [10], [34]. Particular attention should be given to concise wording and consistent grammatical format [10]. Additionally, at every stage of Q-sample development, the research group should reflect on the relevance and adequacy of the statements in relation to the research question [30]. The endpoints of the interval scale should also be defined. In our case, we used “strongly agree” (+3) to “less agree” (-3). Due to the sensitive nature of the research topic, we deliberately avoided the commonly used continuum from “most agree” (+3) to “most disagree” (-3), which is suggested in the literature [5], [10]. Accordingly, the statements were reviewed for their suitability with respect to the scale. The “condition of instruction” was formulated based on the research question, in turn ensuring that the statements corresponded appropriately [30] (see attachment 1 , point B). As a clarifying side note, in the actual study, participants first pre-sorted the statements into three categories (strongly agree (+3 to +1), agree (0), less agree (-3 to -1)). This was intended to reduce the complexity of the statement catalog and facilitate the sorting process. Only then did participants sort the pre-sorted statements into the normal distribution grid. However, they retained the option to reassign statements during this process. Therefore, an item first sorted as “less agree” could still be sorted as “strongly agree” in the final distribution. 

After step 5, our revised Q-sample consisted of 54 statements.

### Evaluating the Q-sample

#### Step 6 – Expert evaluation

To ensure content validity, a panel of experts can be assembled. Experts can contribute to quality assurance when they are meaningfully involved in item development. The expert panel should be diverse and include experts from different fields, such as content and methodological experts [40], [41]. In our study, we invited five experts to participate via personal invitation and snowball sampling: three palliative care physicians (content experts) and two educational scientists with experience conducting Q and survey studies (methodological experts). The panel evaluated the 54 statements of the preliminary Q-sample using a framework adapted from scaled tests, following four criteria:

1. comprehensibility;
2. unambiguousness;
3. distinctiveness; and
4. lack of reference [34].

There was also space for free-text comments (see attachment 1 , point A). The three palliative care physicians evaluated the representativeness and completeness of the Q-sample to ensure that the full range of viewpoints on the study subject was captured by the statements [30]. A Q-method expert reviewed the statements’ usability and provided guidance on Q-sample statement construction [25]. A quantitative medical education researcher evaluated the Q-sample regarding comprehensibility and wording. Finally, all experts were asked to assess the coverage and balance of the Q-sample by answering three open-ended questions:

1. Is a dying and death-specific topic or a particular statement missing in the Q-sample? (This question must be adapted to the respective research objective)
2. Are the topics adequately represented in the Q-sample, or is one topic given too many or too few statements? If so, which one?
3. Are there any other comments on the Q-sample in general?

The open-ended questions supported the assessment of the comprehensiveness of our Q-sample. Based on the expert evaluation, several statements were removed. Reasons for removal included redundancy, lack of reference to the research question, overlap, lack of distinctiveness, direct counterstatements, etc. As a result, the number of statements was reduced from 54 to 42 (see attachment 1 , point A).

#### Step 7 – Piloting the Q-sample

The final step involves piloting the Q-sample to evaluate how the statements perform during the Q-sorting process [42]. For this purpose, representatives of the target group (in our case, five medical students in their final year and three assistant physicians) were asked to sort the Q-sample into a normal distribution grid. The pilot was conducted by a single researcher from our team, who reminded participants to comment on their experience but refrained from asking leading questions. The process of “thinking aloud” [43], in which participants verbalize their spontaneous thoughts while sorting the Q-sample, can be encouraged to gain insight into the cognitive process of Q-sorting and to identify potential challenges. Participants received written instructions about the rank-ordering procedure. After completing the sorting process, they were invited to share their experience and offer suggestions for improvements to the sorting design or individual statements. Their feedback was documented using a short, informal protocol. Based on this feedback, the research group was able to re-evaluate the performance of the statements, the clarity of the sorting instructions, and the ease of use of the (online) Q-sorting tool (FlashQ; [44]). In our case, minor word edits were made to 13 statements, while 29 statements remained unchanged (for the final Q-sample, see attachment 1 , point A).

## Discussion

### The methodological strength of our approach 

The present paper outlines a seven-step approach to Q-sample design based on interview data. Our approach has several strengths that enhance its applicability in medical education research. First, we populated the concourse in a systematically and methodologically sound manner, ensuring that the wide range of viewpoints on the research topic was captured [45]. Thereby, we demonstrate how the results of a qualitative study can be used to design a Q-sample. Drawing on the findings of an interview study to develop the concourse proved valuable for several reasons: it ensured methodological rigor through the systematic identification of themes and sub-themes, and it contributed to the efficient use of research resources. Moreover, the interview study enabled a deeper understanding of the study subject matter, thus helping to ensure the Q-sample was both comprehensive and aligned with the current state of research. Given that participant characteristics were similar in both the interview and the Q-study, we assumed that the themes and sub-themes from the interview study accurately reflected the reference world of the Q-study participants. To inductively identify themes and sub-themes in the interview data, we applied a QCA approach. In addition to QCA, other qualitative methods, such as thematic analysis [46] would also be suitable for systematically identifying themes and sub-themes in (interview) data.

Paige and Morin recommend visualization tools to transfer qualitative research findings into a concourse [25]. We built on this suggestion by visualizing the relevant perceptions from the interviews using concept maps as part of the Q-sample design process. However, the mapping technique served a broader purpose beyond mere visualization. It enabled us to gain an overview of the relevant themes and perceptions, uncover connections between them, and develop a deeper understanding of the subject matter. As such, it served as a filtering tool for identifying relevant viewpoints early in the Q-sample design. This approach offers an innovative and promising alternative to Fisher’s balanced block design [7].

The literature provides limited guidance on how to select a representative Q-sample from the concourse [9], [22], [25]. In particular, a comprehensive overview of the guidelines for assembling a Q-sample is lacking. In this paper, we offer a clear set of inclusion (e.g., relevance of the topic, consistency with previous research, and diversity of opinions) and exclusion criteria (e.g., lack of coverage or overlapping perceptions, narrow topic) to support a systematic selection of relevant perceptions [10]. Several studies highlight the importance of using participants’ language to develop a Q-sample that authentically reflects their lived experiences and perceptions [26], [39]. We also provide practical guidance on converting quotations into statements. Two approaches are presented to address the challenge of breaking down complex quotations containing multiple perceptions into individual, single-idea statements.

An important issue in developing a Q-sample is how to balance the number of statements across (sub-)themes. The present Q-sample was developed following an unstructured approach, resulting in an unequal number of statements per (sub-)theme. Nonetheless, we succeeded in balancing the overall content of the Q-sample, guaranteeing that the included statements widely represented the concourse without overlap or overemphasis of certain (sub-)themes. It would be highly interesting to compare our approach with other methods, such as AI-based techniques or Fisher‘s balanced block design, in the future.

Expert knowledge can be applied in different ways within a Q-study – for instance, Kirschbaum et al. leveraged expert knowledge to select the Q-sample [15]. Involving experts from different backgrounds helps ensure a comprehensive Q-sample [15], [25]. Content experts can verify whether the Q-sample covers all relevant aspects and appropriately represents the concourse [15]. Including methodological experts serves as a quality control or second check on item wording [25]. When selecting experts, we followed Kirschbaum’s recommendations, using personal invitations and snowball sampling [15]. The experts we initially invited recommended additional experts. It is essential to clearly define the experts’ tasks and how their opinions will be evaluated. Therefore, we developed a framework containing four criteria adopted from scaled tests [34]. We added three additional open-ended questions to ensure the Q-sample adequately reflects the full range of viewpoints on the study subject. Experts should be consulted only when item developers cannot close knowledge gaps independently [34]. Contradictory expert opinions must be dealt with critically. There is a risk that item developers will override expert opinions, rendering their contribution obsolete [34]. In cases of contradictory opinions, a second expert evaluation round is recommended [34]. The removal of 12 statements based on expert evaluation underscores the importance of this step and the advantage of using a structured framework. 

The literature emphasizes the importance of piloting a Q-sample and advises that participants be clearly instructed in the rank-ordering procedure [25]. Here, we emphasize the importance of gathering additional information about the Q-sample’s performance, for example, through interviews or the thinking-aloud method. Thinking-aloud provides a deeper understanding of particular challenges and inconsistencies within the Q-sample. 

To summarize, in this paper, we present a structured guide to designing a Q-sample, describing a seven-step approach that integrates a mapping technique and offers detailed considerations for each step. In particular, the explicit decision-making criteria and the use of evaluative frameworks add methodological rigor to our approach, helping to ensure the quality of the Q-sample and minimize researcher bias [15]. The next step is to apply this approach and evaluate how the Q-sample performs in a study, to determine if this method is suitable for deriving a valid Q-sample. The study described here is ongoing, with the finalized Q-sample being used in two cohort groups: students and assistant physicians. In a follow-up publication, we will report our findings and share our experiences with the Q-sample. In parallel, we encourage other researchers to adopt this approach and offer ideas for further refinement. Finally, it would be highly valuable to compare this seven-step method with alternative strategies for Q-sample development.

### Lessons learned 

Several challenges need to be addressed. Our complex research question resulted in an extensive catalog of statements with convoluted content, which proved difficult to reduce and standardize. Accordingly, we suggest focusing the research question on a less complex topic to reduce the number of statements and make them more tangible. Specifically, we recommend dividing complex topics into subtopics and exploring them individually (for example, in our case, communication regarding dying and death). One key aspect is balancing the number of statements per (sub-)theme. We based this number on the relevance of each theme in the interviews, which led to an unequal distribution of statements. From a qualitative perspective, we understand balancing as achieving a content-based equilibrium, guided by the relevance and depth of themes, rather than enforcing a numerical equalization of items per theme. The aim is to prevent an over-representation of less important topics. However, this approach may also lead to an over- or underrepresentation of certain topics. Therefore, researchers should critically reflect on this step, discuss which topics are more or less important, and ensure transparency and comprehensibility. Our final Q-sample included statements assessing either the importance or difficulty of tasks. The dimension “important” was intended to capture the professional perspective on specific tasks, while the dimension “difficult” aimed to reflect the emotional and cognitive experience of these tasks on a personal level. The goal was to explore the interaction between these professional and personal dimensions. However, participants found it challenging to sort statements with different qualifiers (importance/ difficulty) side by side. To address this, we recommend focusing on a single dimension, such as either importance or difficulty.

During the piloting of the Q-sample, we observed that generic statements (i.e., overly broad or unspecific) yielded limited insights, as they were predominantly assigned to the extreme ends of the distribution. For example, the statement “as a physician, I must protect the patient’s dignity” was consistently ranked highly due to its general nature. To avoid this, attention should be paid to clear and focused wording.

In addition, the limitations associated with concept mapping should be acknowledged. To minimize participant bias in the previous study, we purposefully selected a diverse sample consisting of students and early-career physicians from various medical fields. We also consulted relevant literature when constructing the concept maps to ensure a comprehensive perspective on the research subject. To mitigate researcher bias and “control subjectivity” [47], two researchers collaborated on the mapping process. Nevertheless, the inherent subjectivity of concept mapping remains a potential limitation and should be recognized as such.

## Conclusion

Q enables the study of subjectivity by identifying attitudinal patterns across groups and individuals. It allows for the investigation of controversial topics with clear emotional and moral dimensions, which are common in health education research [9]. Given the method's potential, this paper describes a seven-step approach to designing a Q-sample based on interview data. We introduce an innovative element by using a mapping technique to select relevant perceptions and statements from the concourse. We provide a detailed overview of the criteria for constructing and refining the Q-sample. Furthermore, we contribute to the existing literature by demonstrating the practical value of following a sequential approach in Q-sample development. We also shed light on the iterative nature of Q-sample construction and showcase its potential applications in medical education research. As such, this paper serves as a *how-to manual* for developing a Q-sample based on interview data that adheres to the principle of self-reference.

## Authors’ ORCIDs

- Nana Jedlicska: [0000-0001-8229-7845]
- Pascal O. Berberat: [0000-0001-5022-5265]
- Kristina Schick: [0000-0002-4819-4604]

## Competing interests

The authors declare that they have no competing interests. 

## Supplementary Material

Statement evaluation questionnaire for the expert evaluation and final Q-sample (A)

## Figures and Tables

**Table 1:**
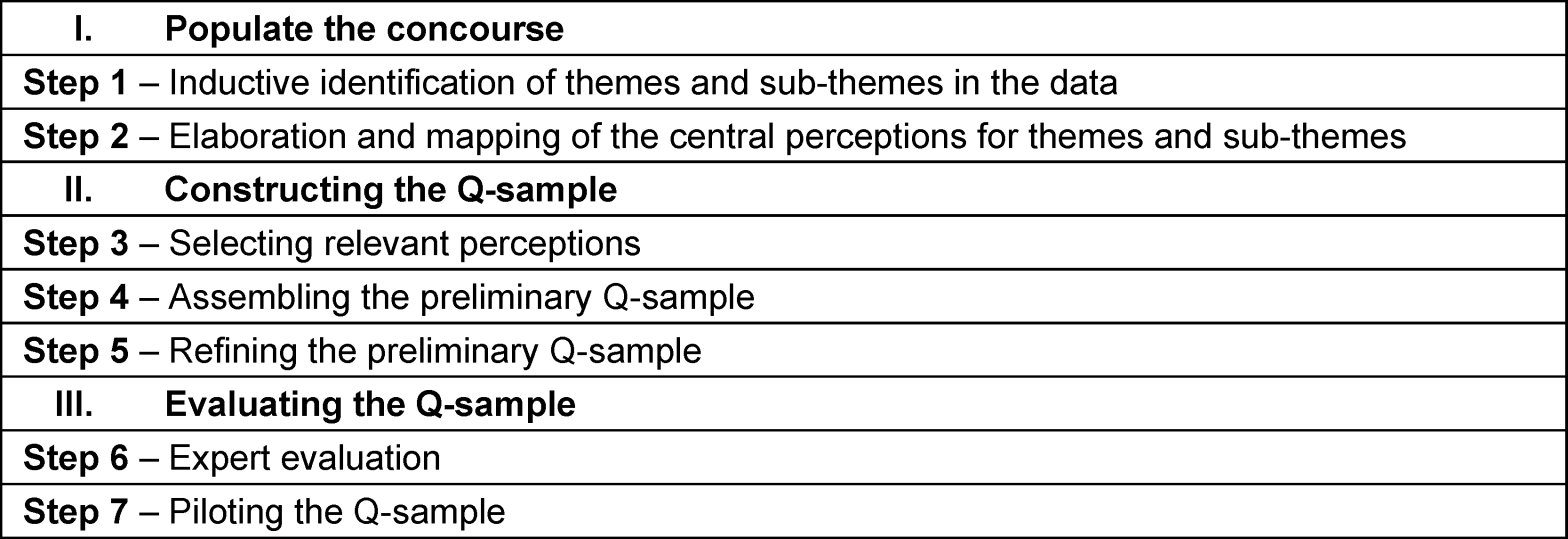
Steps in the Q-sample design
