# Supplementary material for: How to design a Q-sample: A seven-step approach based on interview data
Source: GMS J Med Educ. 2026 Jan 15;43(1):Doc8. doi: 10.3205/zma001802 (PMC12875056; doi:10.3205/zma001802)
Supplement: Statement evaluation questionnaire for the expert evaluation and final Q-sample (A) [file JME-43-8-s-001.pdf]

Attachment 1: Statement evaluation questionnaire for the expert evaluation and final Q-sample (A)

Preliminary Q-sample	Final Q-sample
**1. Distancing**	
1.1. As a physician, I am uncertain about where I need to draw the line between empathy and distance.	1. As a physician, I am uncertain where to draw the right line between empathy and distance.
1.2. It is more difficult for me as a physician to maintain distance the closer the connection is with the patient.	2. It is more difficult for me as a physician to maintain distance the closer the connection is with the patient.
1.3. As a physician, I must set a limit on how much I empathize with patients.	*repetitive statement to 1.1*
1.4. As a physician, I worry about becoming desensitized with increasing experience.	3. As a physician, I worry about becoming desensitized with increasing experience.
1.5. As a physician, I need to distance myself from the patient to fulfill my medical duties.	4. As a physician, I need to distance myself from the patient to fulfill my medical duties.
1.6. As a physician, I only have a limited control over how much I empathize with patients.	5. As a physician, I cannot always control how deeply I empathize with patients.
1.7. As a physician, I feel guilty when a patient dies in my care.	6. As a physician, I feel guilty when a patient dies in my care.
1.8. With increasing experience, it becomes easier for me as a physician to deal with dying and death.	7. With increasing experience, it becomes easier for me as a physician to deal with dying and death.
1.9. As a physician, I need to distance myself from the patient to protect myself.	8. As a physician, I need to distance myself from the patient to protect myself.
**2. Keeping in control**	
2.1. It is more difficult for me as a physician when a patient passes away unexpectedly.	9. It is more difficult for me as a physician when a patient passes away unexpectedly.
2.2. As a physician, I am not allowed to lose control of my emotions in front of the patient.	10. As a physician, I am not allowed to lose control of my emotions in front of the patient.
2.3. It is more difficult for me as a physician when a young patient passes away.	11. It is more difficult for me as a physician when a young patient passes away.
**3. Thoughtful care**	
3.1. As a physician, I must also provide psychosocial support to patients.	12. As a physician, I must also provide psychological support to patients.
3.2. As a physician, I must also take an interest in the person of the patient.	*missing reference*
3.3. As a physician, I must take time for the patients.	13. As a physician, I must take time for the patients.
3.4. As a physician, I must preserve the dignity of the patient.	14. As a physician, I must preserve the dignity of the patient.
3.5. The working conditions make it difficult for me as a physician to be there for the patients even more.	15. The working conditions make it difficult for me as a physician to be there for the patients.
3.6. As a physician, I must show empathy.	16. As a physician, I must show empathy.
3.7. As a physician, I must build a trusting relationship with the patient.	17. As a physician, I must build a trusting relationship with the patient.

Attachment 1 to Jedlicska N, Lichtenberg S, Berberat PO, Schick K. *How to design a Q-sample: A seven-step approach based on interview data.* GMS J Med Educ. 2026;43(1):Doc8. DOI: 10.3205/zma001802

**4. Making decisions and taking responsability**	
4.1. As a physician, I bear responsibility for the life and death of the patient.	18. As a physician, I am responsible for the life and death of the patient.
4.2. It is difficult for me as a physician to have to stand by my therapeutic decisions.	19. It is difficult for me as a physician to have to stand by my therapeutic decisions.
**5. Communication**	
5.1. As a physician, I must discuss the medical situation with the patient openly.	20. As a physician, I must honestly discuss the medical situation with the patient.
5.2. As a physician, I must inform the patient in good time if their condition is serious.	21. As a physician, I must inform the patient in time if their condition is serious.
5.3. It is difficult for me as a physician to openly tell the patient that they are dying.	22. It is difficult for me as a physician to openly tell the patient that they are dying.
5.4. As a physician, I am not allowed to discourage the patient.	23. As a physician, I am not allowed to discourage the patient.
5.5. As a physician, I must inquire about the patient's wishes.	24. As a physician, I must actively inquire about the patient's wishes.
5.6. For me as a physician, it is relieving when I have spoken openly with the patient.	*counterstatement to 5.1*
**6. Caring for relatives**	
6.1. For me as a physician, the death of the patient takes on a positive light if I feel that I have taken good care of the relatives.	25. For me as a physician, the death of the patient takes on a positive light if I feel that I have taken good care of the relatives.
6.2. As a physician, it is my duty to give the relatives the opportunity to say goodbye properly.	26. As a physician, it is my duty to give the relatives the opportunity to say goodbye properly.
6.3. As a physician, it is my responsibility to prepare the family members for the death of the patient.	27. As a physician, it is my responsibility to prepare the family members mentally for the death of the patient.
6.4. As a physician, I must be available as a contact person for the relatives.	28. As a physician, I must be available as a contact person for the relatives.
6.5. For me as a physician, the death of the patient would be easier without the relatives.	29. For me as a physician, the death of the patient would be easier without the relatives.
**7. Allowing death**	
7.1. As a physician, I believe that one does not always have to do everything until the end.	*repetitive statement to 7.5*
7.2. As a physician, I must also grapple with not being able to cure.	30. As a physician, I must also grapple with the not being able to cure.
7.3. As a physician, I must consider whether the therapy does more harm than good.	31. As a physician, I must consider whether the therapy does more harm than good.
7.4. It is difficult for me as a physician to deal with my own expectations of healing.	32. It is difficult for me as a physician to deal with my own expectations of healing.
7.5. As a physician, I must eventually find the point where I let the patients go.	33. As a physician, I must eventually find the point where I let the patients go.

7.6. As a physician, I have a calmer conscience when I have exhausted all medical options.	34. As a physician, I have a calmer conscience when I have exhausted all medical options.
7.7. As a physician, I have doubts when the responsible doctors do not allow patients to pass away.	*counterstatement to 7.6*
**8. Enabling a beautiful death**	
8.1. As a physician, it is my responsibility to create a pleasant setting at the end of life.	35. As a physician, it is my responsibility to create a pleasant environment at the end of life.
8.2. As a physician, it is my duty to alleviate suffering.	36. As a physician, it is my duty to alleviate suffering.
8.3. As a physician, it is my duty to respect the wishes of the patient.	37. As a physician, it is my duty to respect the wishes of the patient.
8.4. As a physician, it is my duty to alleviate the patient's fear of death.	38. As a physician, it is my duty to alleviate the patient's fear of death.
8.5. It is more challenging for me as a physician when there is no advance directive.	39. It is more challenging for me as a physician when there is no advance directive.
**9. The image of dying & death**	
9.1. As a physician, I believe that my attitude towards dying and death influences my interaction with patients.	40. As a physician, I believe that my attitude towards dying and death influences my interaction with patients.
9.2. As a physician, I believe that death is not the worst outcome.	41. As a physician, I believe that death is not the worst outcome.
9.3. As a physician, I believe that dying requires individualized care.	42. As a physician, I believe that dying requires individualized care.
9.4. As a physician, I view death more pragmatically because I can explain death pathophysiologically.	*overlapping statement to 9.2*
9.5. As a physician, I believe that death is a part of life.	*overlapping statement to 9.2*
**10. Attitudes on and resolutions for life**	
10.1. As a physician, encountering dying and death has changed my perspective on life.	*missing reference*
**11. Wishes for one’s own dying and death**	
11.1. As a physician, I wish to have the right to die autonomously.	*missing reference*
11.2. As a physician, I wish to have sufficient time for my own death.	*missing reference*
11.3. As a physician, I desire a painless death.	*missing reference*
11.4. As a physician, I do not wish to die in a hospital.	*missing reference*

Each statement was evaluated based on the following scale:

- ☐ not distinguishable/ duplicate
- ☐ incomprehensible
- ☐ other[1]
- ☐ ambiguous
- ☐ too general/ lacking reference

Evaluation of coverage and balance

1. Is a dying and death-specific topic or a particular statement missing in the Q-sample?
2. Are the topics adequately represented in the Q-sample, or is one topic given too many or too few statements? If so, which one?
3. Are there any other comments on the Q-sample in general?

[1] These criteria were included in the original expert questionnaire as options to be selected after each question. However, they are not listed in the current presentation due to space reasons.

Attachment 1: Sorting instruction for the participants to sort Q-set (B)

1. At the beginning, you see various statements: To what extent can you identify with the statements?

   Refer explicitly to your personal experiences with dying and death. Put yourself in the role of a physician if you are not yet licensed.

   Please read the statements carefully and divide them evenly between the three categories:

   - Statements you strongly agree with (+3 to +1)
   - Statements you agree with (0)
   - Statements you agree with less (-3 to -1)

   You can either drag the cards into the appropriate field or use the 1, 2 or 3 keys on your keyboard.

2. After the pre-sorting, please order the statements into the normal distribution grid from +3 to -3. You can change the pre-sorting afterwards (you can sort a statements of “agree with less” still to “strongly agree with” and vice versa.

3. Please check your final sorting and interchange statements if necessary.
